# Aeromonas hydrophila Necrotizing Soft-Tissue Infection With Acute-on-Chronic Calcaneal Osteomyelitis Following Freshwater Exposure in a Patient With Poorly Controlled Diabetes Mellitus

**DOI:** 10.7759/cureus.116193

**Published:** 2026-09-13

**Authors:** Adrian De La Rosa, Ana L Rivera Reyes, Ruben H Alvarez Cobian

**Affiliations:** 1 Internal Medicine, Ponce Health Sciences University, Ponce, PRI; 2 Internal Medicine, Hospital de la Concepción, San German, PRI

**Keywords:** aeromonas hydrophila, diabetic foot infection, freshwater exposure, necrotizing fasciitis, osteomyelitis, soft tissue infection

## Abstract

*Aeromonas hydrophila* is a gram-negative bacterium found in freshwater environments that can cause rapidly progressive necrotizing soft-tissue infections (NSTIs), particularly in patients with predisposing host factors such as diabetes mellitus. The organism rarely affects deeper structures such as bone, which can make diagnosis and treatment more difficult. We report the case of a 68-year-old man with poorly controlled type 2 diabetes mellitus, heart failure with reduced ejection fraction, and prior calcaneal trauma who developed an NSTI with acute-on-chronic calcaneal osteomyelitis after stagnant rainwater entered a closed shoe. Symptoms began later that day and progressed over the next five days, culminating in a limb-threatening infection with sepsis documented by the treating teams and requiring urgent surgical intervention. Operative abscess cultures obtained during incision and drainage grew *A. hydrophila* complex, while calcaneal bone biopsy demonstrated osteonecrosis with acute osteomyelitis; the pathology report also listed a methicillin-resistant *Staphylococcus aureus* marker. The patient was managed with surgical debridement and antimicrobial therapy directed by Infectious Diseases, ultimately receiving intravenous ceftriaxone and levofloxacin to complete a six-week course. This case underscores the importance of considering environmental exposures, obtaining early surgical consultation, and using operative microbiology and pathology to guide management of rapidly progressive soft-tissue infections.

## Introduction

Necrotizing soft-tissue infections (NSTIs) are serious surgical emergencies characterized by rapid tissue destruction and high mortality [1]. A nationwide Danish cohort reported an incidence of 1.99 per 100,000 person-years and a 30-day mortality of 19.4%, with diabetes mellitus as the most common comorbidity [2]. *Aeromonas* species are uncommon causes of NSTI; in a recent 14-year retrospective series of 105 patients with *Aeromonas* infections, necrotizing fasciitis occurred in only two cases, underscoring the rarity of this presentation [3]. *Aeromonas* species, including *Aeromonas hydrophila*, are gram-negative aquatic organisms that can cause severe skin and soft-tissue infections, particularly following water exposure [3,4]. Diabetes mellitus is among the frequently reported comorbidities in affected patients [3]. *A. hydrophila* possesses multiple virulence determinants characterized in necrotizing fasciitis-associated strains, including the aerolysin-related cytotoxic enterotoxin Act and type III and VI secretion-system components and effectors [5]. In murine models, ExoA has been implicated in local tissue-barrier destruction and bacterial dissemination [6]. Concomitant calcaneal osteomyelitis in a patient with an *Aeromonas* NSTI is uncommon and can create additional diagnostic and therapeutic challenges, particularly when the affected bone has pre-existing post-traumatic and post-infectious changes. Different microbiologic findings may also be present in adjacent soft tissue and bone compartments. Here, we present a case of *A. hydrophila* NSTI with acute-on-chronic calcaneal osteomyelitis following freshwater exposure and discuss the diagnostic and management implications for clinicians who may not routinely encounter this presentation.

## Case presentation

A 68-year-old man with poorly controlled type 2 diabetes mellitus, heart failure with reduced ejection fraction (HFrEF) (20%-25%) with a single-chamber implantable cardioverter-defibrillator, atrial fibrillation treated with anticoagulation, hyperlipidemia, hypertension, gout, obesity (BMI: 30.1 kg/m^2^), and chronic alcohol use presented to the emergency department with worsening pain, swelling, erythema, and purulent drainage of the left lower extremity. Poor glycemic control was supported by the patient's report of home glucose values frequently approaching 600 mg/dL after a hospitalization two months earlier for drainage of a left axillary abscess, together with an admission serum glucose of 213 mg/dL and marked glucosuria (≥1000 mg/dL).

The patient had sustained a left calcaneal fracture 19 years earlier that required surgical fixation and was subsequently complicated by osteomyelitis requiring treatment and surgical debridement. He reported that the prior osteomyelitis had resolved, although he continued to experience intermittent heel pain and a morning limp. He later explained that these chronic symptoms initially led him to attribute the new pain to his prior calcaneal condition, contributing to delayed medical evaluation.

Five days before hospital presentation, the patient was emptying an outdoor garbage bin containing rainwater that had remained stagnant for approximately 10 days when the water spilled into his closed shoe and soaked his sock. The shoe remained wet for several hours. Later that afternoon, he noticed pruritus followed by marked swelling, erythema, dark discoloration, and superficial abrasions corresponding to areas of shoe friction. Over the following five days, the pain became pulsatile and progressively debilitating, swelling and warmth increased, and he eventually became unable to ambulate and remained bedbound. He also reported intermittent fever, chills, dizziness, malaise, and generalized weakness. On the day of presentation, ambulation into the emergency department was followed by purulent drainage from the affected heel/ankle region.

At the initial emergency department evaluation in November 2025, his temperature was 36.9°C, heart rate 94 beats/min, respiratory rate 18 breaths/min, blood pressure 119/74 mmHg, and oxygen saturation 96% on room air. He was alert and oriented, without focal neurologic deficits. Serum lactate was 1.3 mmol/L, and creatinine was 1.18 mg/dL, with preserved renal function. By the morning of hospital day 1, he had developed fever to 38.7°C, tachycardia to 114 beats/min, and tachypnea to 24 breaths/min in the setting of leukocytosis and a severe limb-threatening infection. Sepsis secondary to the left lower-extremity infection was documented by both the Internal Medicine and Infectious Diseases services.

Physical examination demonstrated diffuse erythema, swelling, and warmth extending from the foot to the distal two-thirds of the lower leg, with tense circumferential edema, bullae, violaceous discoloration, and purulent drainage (Figure 1). Pain extended proximally toward the knee. The Infectious Diseases examination additionally documented a fluctuant lateral foot lesion with an open bulla. Wound Care described a malodorous left lateral heel ulcer measuring 1.3 x 1.2 x 0.3 cm.

**Figure 1:**
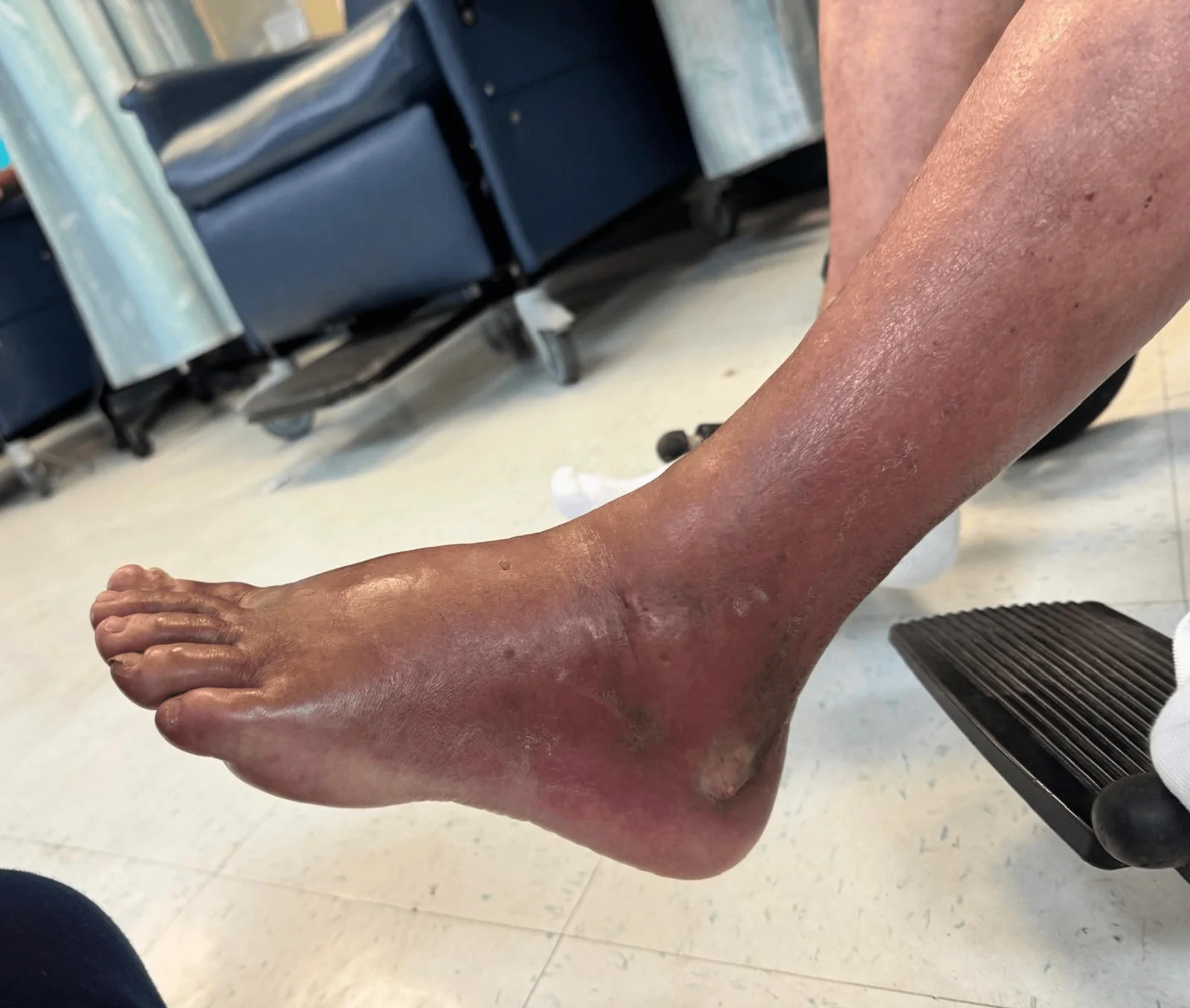
Initial Presentation of Left Lower Extremity Necrotizing Soft-Tissue Infection Initial presentation of the left lower extremity in November 2025 (day of admission), demonstrating diffuse erythema, tense edema, bullae, and violaceous discoloration of the left foot and distal lower leg in the setting of suspected necrotizing soft-tissue infection.

Admission laboratory studies demonstrated leukocytosis (14.13 x 10^3^/uL), C-reactive protein 35.7 mg/dL, erythrocyte sedimentation rate 48 mm/h, and procalcitonin 1.34 ng/mL. The admission Laboratory Risk Indicator for Necrotizing Fasciitis (LRINEC) score was 7. Because LRINEC should not be used in isolation to confirm or exclude NSTI, the score was interpreted together with the rapidly progressive clinical findings and imaging [7]. The individual LRINEC components are summarized in Table 1.

**Table 1:** Admission Laboratory Values and LRINEC Score Components LRINEC, Laboratory Risk Indicator for Necrotizing Fasciitis.

Parameter	Admission value	LRINEC points
C-reactive protein	35.7 mg/dL (357 mg/L)	4
White blood cell count	14.13 x 10^3^/uL	0
Hemoglobin	10.4 g/dL	2
Sodium	136 mmol/L	0
Creatinine	1.18 mg/dL	0
Glucose	213 mg/dL	1
LRINEC score (total)		7

Plain radiographs of the left ankle demonstrated posterior calcaneal destruction with periosteal reaction concerning for osteomyelitis, together with soft-tissue swelling and gas in the leg (Figure 2), raising concern for a gas-forming necrotizing infection. Bilateral arterial Doppler studies showed no significant arterial obstruction. Although the radiology report suggested MRI for further characterization of the calcaneus, no MRI was obtained before surgery. Given the rapid clinical progression, soft-tissue gas on radiography, and high clinical suspicion for NSTI, urgent operative management proceeded without delaying for advanced imaging.

**Figure 2:**
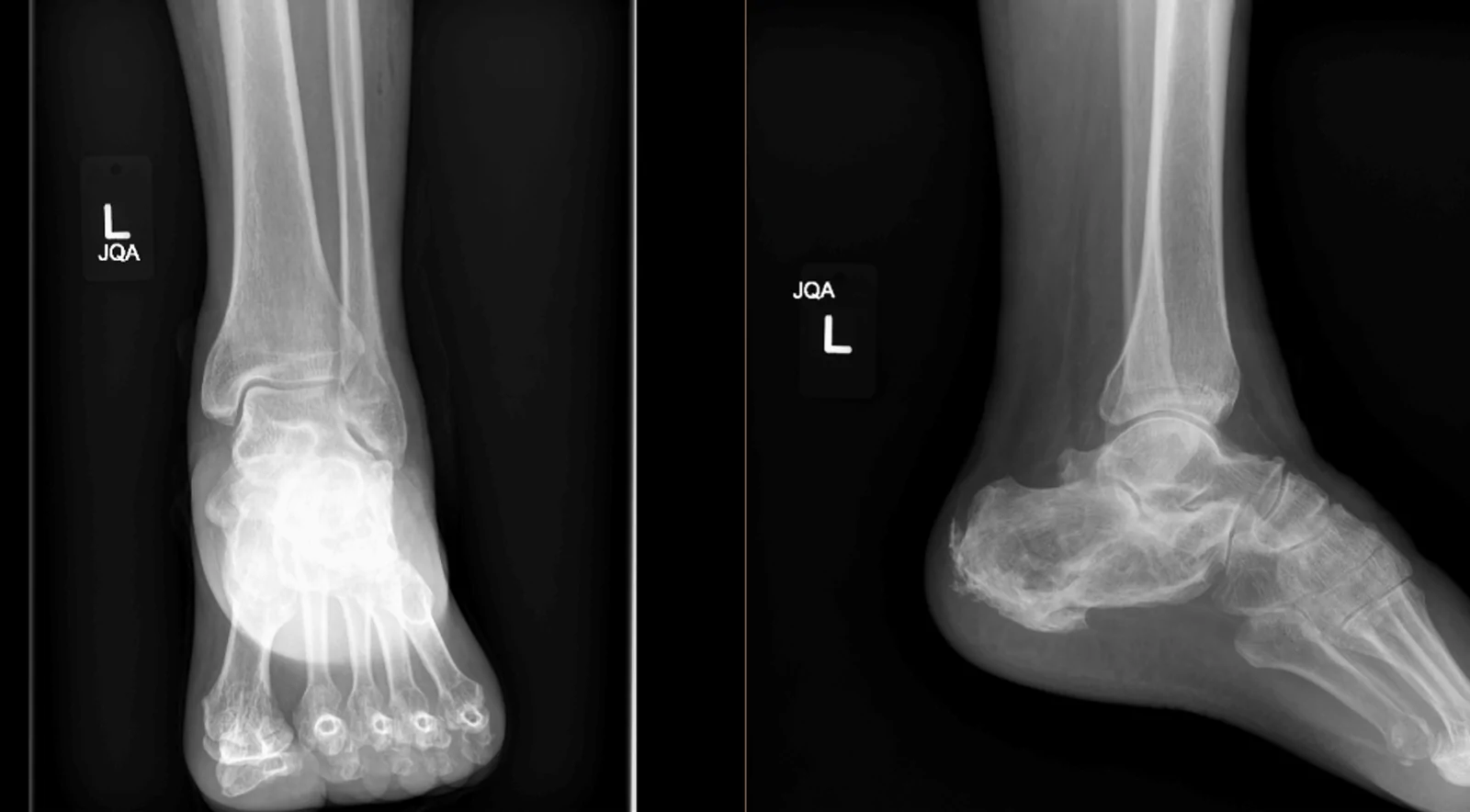
Plain Radiographs Demonstrating Calcaneal Osteomyelitis and Associated Soft-Tissue Gas Plain radiographs of the left ankle obtained on November 2025 (day of admission), demonstrating posterior calcaneal destruction with periosteal reaction concerning for osteomyelitis, together with associated soft-tissue gas raising concern for a gas-forming necrotizing infection.

Empiric intravenous piperacillin-tazobactam and vancomycin were initiated on admission. After Infectious Diseases evaluation and concern for a severe limb-threatening necrotizing infection with soft-tissue gas, clindamycin was added. General Surgery evaluated the patient the same morning and proceeded to the operating room that day for incision and drainage of the left foot abscess and open calcaneal bone biopsy. The operative report documented removal of all purulent and necrotic material, irrigation of the wound, and open packing after biopsy.

The operative left foot abscess culture grew *A. hydrophila* complex. Infectious Diseases documented the isolate as pan-susceptible and later specifically noted susceptibility to ciprofloxacin; the complete organism-specific susceptibility panel was not available in the records reviewed for this report. Three blood cultures remained negative after five days. Vancomycin and clindamycin were discontinued, piperacillin-tazobactam was continued, and intravenous ciprofloxacin was added.

Histopathologic examination of the calcaneal bone biopsy demonstrated osteonecrosis with acute osteomyelitis. The pathology report additionally listed a methicillin-resistant *Staphylococcus aureus* (MRSA) marker. A separate bone susceptibility report was not available in the reviewed record, and on December 4, the Internal Medicine note documented that MRSA susceptibility remained unavailable, while outpatient antibiotic recommendations were pending. The final discharge regimen was intravenous ceftriaxone 2 g daily plus levofloxacin 750 mg daily to complete a 42-day course, as directed by the treating team. The patient demonstrated gradual clinical improvement after debridement and antimicrobial therapy. By postoperative day 3, the surgical wound measured approximately 4 × 6 cm and demonstrated viable tissue with developing granulation and residual devitalized areas, without recurrent suppuration or foul odor (Figure 3).

**Figure 3:**
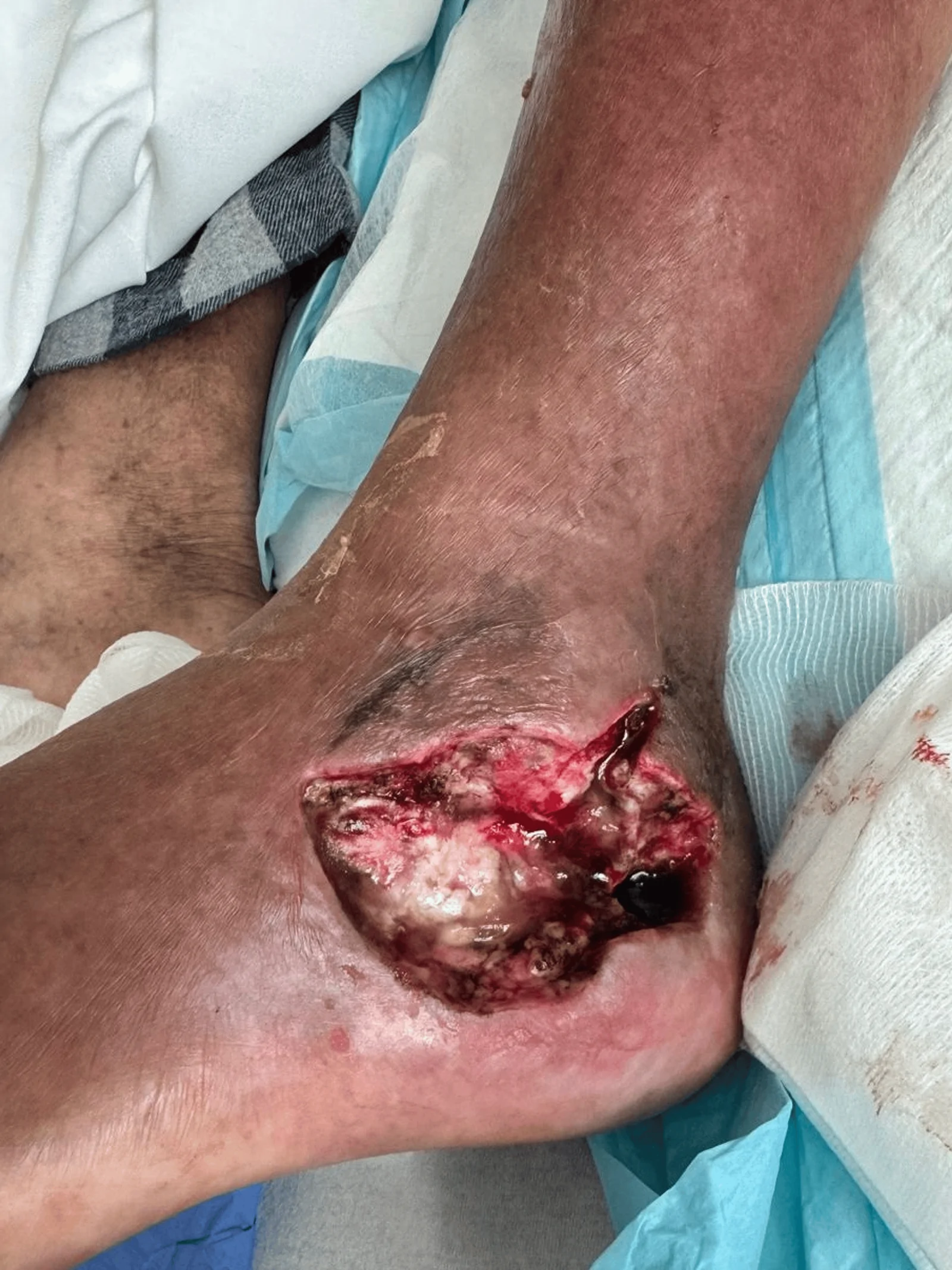
Post-debridement Appearance of the Left Lateral Heel Following Surgical Incision and Drainage Post-debridement appearance of the left lateral heel in November 2025 (postoperative day 3), demonstrating a large residual soft-tissue defect with exposed subcutaneous tissue, developing granulation tissue, and areas of residual devitalized tissue following surgical incision and drainage.

On December 3, two days before discharge, Wound Care documented a 3.8 x 5.7 x 0.5 cm lateral heel wound with a pink granulating bed, approximately 40% non-viable tissue, mild serous drainage, and no clinical signs of infection (Figure 4). He was discharged on December 5, with a peripherally inserted central catheter, home intravenous antibiotic infusion, and home wound-care support. No outpatient follow-up records were available for this report.

**Figure 4:**
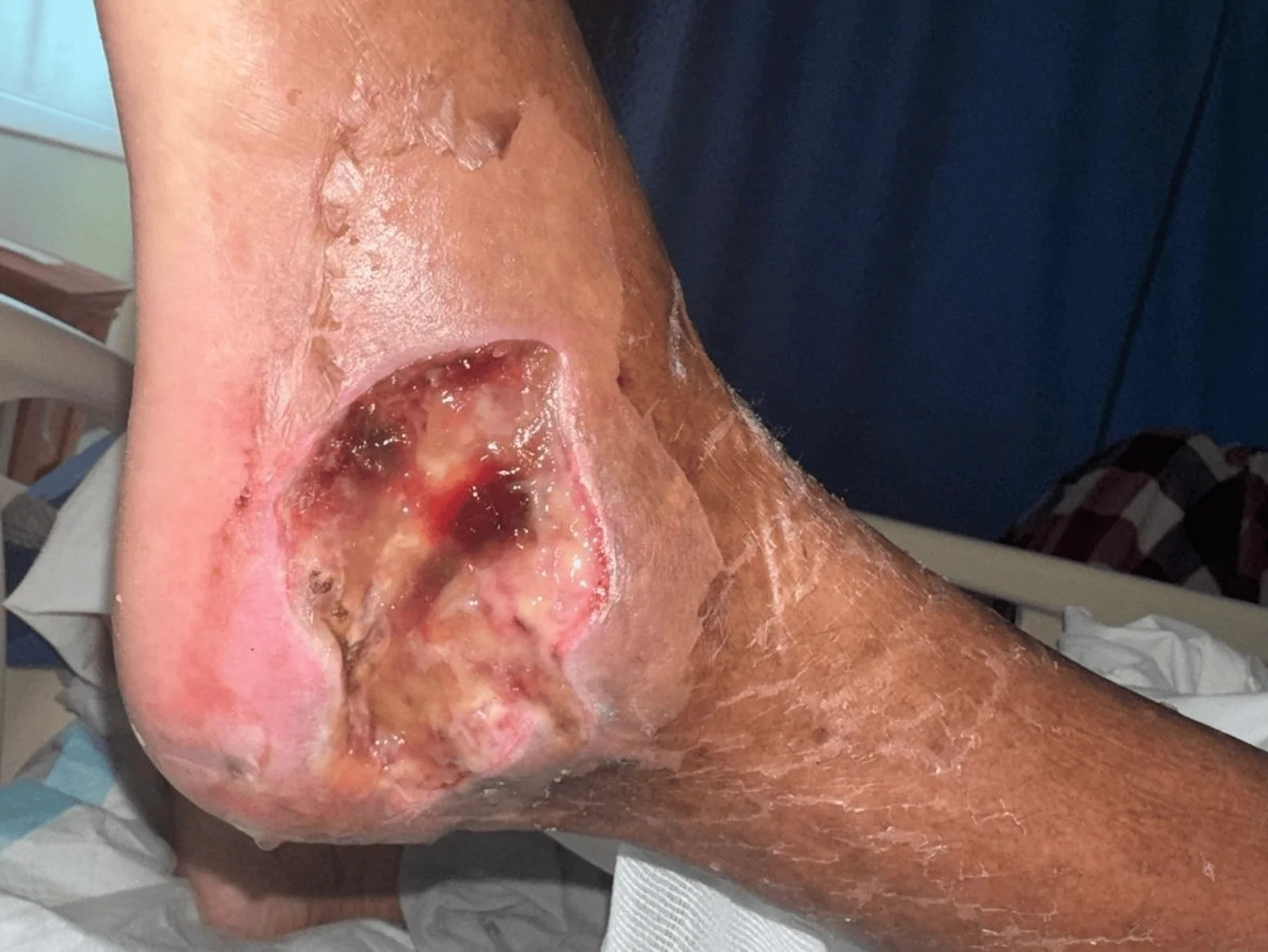
Interval Post-debridement Appearance of the Left Lateral Heel Demonstrating Granulation Tissue Formation Interval post-debridement appearance of the left lateral heel on December 3, 2025, two days before discharge, demonstrating evolving granulation tissue within the residual wound following surgical debridement, ongoing wound care, and antimicrobial therapy.

## Discussion

This case highlights several important considerations in NSTIs caused by *A. hydrophila*, including the value of a detailed environmental exposure history, the impact of medical comorbidities, the importance of early surgical source control, and the challenges created by different microbiologic findings in soft tissue and bone.

History of environmental exposure

Obtaining an environmental exposure history was critical to this patient's diagnosis. *Aeromonas* species are widely distributed in aquatic environments, including freshwater sources, and can cause wound and soft-tissue infections following water exposure [4]. In this case, stagnant rainwater remained trapped inside a closed shoe for several hours, and prolonged moisture together with friction-related abrasions likely facilitated bacterial inoculation through compromised skin. The patient developed local symptoms later the same day. A detailed exposure history is especially important in rapidly progressive soft-tissue infections because recent freshwater contact can narrow the microbiologic differential, inform empiric antimicrobial selection, and heighten concern for aggressive water-associated pathogens.

Impact of concurrent illnesses

This patient's poorly controlled type 2 diabetes mellitus, HFrEF, and obesity increased the complexity of his presentation and treatment. In a nationwide cohort of patients with NSTI, diabetes was the most common comorbidity, affecting 43% of patients [2]. In this patient, poor glycemic control was supported by markedly elevated home glucose readings reported before admission, admission hyperglycemia, glucosuria, and decreased lower-extremity sensation and paresthesias documented on examination. These findings are clinically important because impaired sensation may delay recognition of skin injury, while hyperglycemia can adversely affect host defense and wound healing.

HFrEF of 20%-25% added perioperative and medical complexity, particularly with respect to fluid administration and hemodynamic management. His remote calcaneal fracture and previous postoperative osteomyelitis also complicated interpretation of the new symptoms. The patient had experienced intermittent heel pain for years and initially attributed the new pain to his prior injury, contributing to delayed presentation. Although histopathology in this admission demonstrated acute rather than chronic osteomyelitis, the pre-existing post-traumatic and post-infectious calcaneal changes provide the chronic structural background reflected in the clinical description of acute-on-chronic disease.

Key importance of early surgical management

Timely surgical source control is a major determinant of outcome in NSTI. A systematic review and meta-analysis found lower mortality when initial surgery occurred within six hours of hospital presentation compared with later surgery (19% versus 32%; OR: 0.43, 95% CI: 0.26-0.70) [8]. This data emphasize the need for rapid surgical evaluation once NSTI is suspected. In this case, the patient presented after approximately five days of progressive symptoms and underwent operative debridement on hospital day 1 after evaluation by Infectious Diseases and General Surgery.

Plain radiography can provide an important clue when soft-tissue gas is present, although its sensitivity is limited. A systematic review reported a sensitivity of 48.9% and specificity of 94.0% for plain radiography in NSTI [7]. Accordingly, the absence of gas cannot exclude NSTI, but its presence in a rapidly progressive infection should prompt urgent surgical consultation. In this patient, radiographic soft-tissue gas, bullae, violaceous discoloration, progressive pain, and systemic inflammatory findings supported immediate operative management. MRI had been suggested for further evaluation of osteomyelitis but was not obtained before surgery, appropriately avoiding delay in source control.

Diagnostic workup and antimicrobial therapy

The diagnostic approach appropriately integrated clinical assessment, basic imaging, operative findings, and deep specimen sampling. The LRINEC score of 7 increased concern for NSTI but was not treated as a stand-alone diagnostic test; systematic review data show that LRINEC has insufficient sensitivity to exclude NSTI when clinical suspicion is high [7]. Operative abscess cultures also provided a more clinically meaningful specimen than a superficial wound swab and established *A. hydrophila* complex, as the organism recovered from the soft-tissue infection.

Empiric antimicrobial therapy with vancomycin and piperacillin-tazobactam provided broad coverage consistent with guideline-based management of suspected necrotizing infection [9]. Clindamycin was additionally initiated by the Infectious Diseases team, given concern for a rapidly progressive, gas-forming soft-tissue infection. After the operative abscess culture grew pan-susceptible *A. hydrophila* complex, therapy was adjusted to piperacillin-tazobactam plus ciprofloxacin. *Aeromonas* species are generally susceptible to fluoroquinolones and third-generation cephalosporins [4], although susceptibility may vary and clinically important resistance has been reported, reinforcing the importance of susceptibility-guided antimicrobial therapy [3,4].

The calcaneal biopsy subsequently demonstrated osteonecrosis with acute osteomyelitis and also listed an MRSA marker. The available record did not contain a final MRSA susceptibility panel or an explicit Infectious Diseases explanation reconciling this finding with the definitive discharge regimen. We therefore report the antimicrobial course as documented rather than infer an undocumented rationale. The patient was ultimately discharged with intravenous ceftriaxone and levofloxacin to complete 42 days of therapy after surgical source control. This uncertainty in the retrospectively available microbiology record is an important limitation when interpreting the final antimicrobial selection.

Implications for clinical practice

​This case underscores several points relevant to clinical practice. A seemingly minor environmental exposure, such as stagnant rainwater trapped inside a closed shoe, can provide an important diagnostic clue when soft-tissue symptoms progress rapidly. Poorly controlled diabetes and peripheral sensory deficits may increase vulnerability to skin injury and delay recognition. Pre-existing orthopedic disease can further obscure a new infection when patients attribute progressive pain to chronic symptoms. Finally, prompt multidisciplinary collaboration among Internal Medicine, Infectious Diseases, General Surgery, Wound Care, and Pathology is essential for source control, microbiologic diagnosis, and coordinated antimicrobial management.

## Conclusions

This case illustrates how rapidly *A. hydrophila* can cause a limb-threatening NSTI after seemingly limited freshwater exposure. Stagnant rainwater trapped inside a closed shoe, together with friction-related skin injury and poorly controlled type 2 diabetes mellitus, preceded a rapidly progressive infection requiring surgical source control. The patient's remote calcaneal fracture, previous osteomyelitis, and chronic heel pain also created a diagnostic pitfall by initially providing an alternative explanation for his worsening symptoms. Successful management required detailed exposure history, high clinical suspicion, urgent multidisciplinary evaluation, operative debridement, deep specimen collection, wound care, and repeated adjustment of antimicrobial therapy. The case also demonstrates that advanced imaging should not delay surgical management when the clinical and radiographic findings strongly suggest NSTI. As a single case report with incomplete outpatient follow-up and an unavailable final MRSA susceptibility panel in the reviewed record, these findings are illustrative rather than generalizable and should not be interpreted as establishing an optimal antimicrobial regimen. Nonetheless, the case reinforces the importance of early recognition, source control, and coordinated multidisciplinary care in severe water-associated soft-tissue infection.
